# Predictive modeling of depression and anxiety using electronic health records and a novel machine learning approach with artificial intelligence

**DOI:** 10.1038/s41598-021-81368-4

**Published:** 2021-01-21

**Authors:** Matthew D. Nemesure, Michael V. Heinz, Raphael Huang, Nicholas C. Jacobson

**Affiliations:** 1 Center for Technology and Behavioral Health, Geisel School of Medicine, Dartmouth College, 46 Centerra Parkway, Lebanon, NH 03766 USA; 2 Quantitative Biomedical Sciences Program, Dartmouth College, 1 Medical Center Dr, Lebanon, NH 03766 USA; 3 Dartmouth-Hitchcock Medical Center, 1 Medical Center Dr, Lebanon, NH 03766 USA; 4 Department of Biomedical Data Science, Geisel School of Medicine, Dartmouth College, 1 Medical Center Dr, Lebanon, NH 03766 USA; 5 Department of Psychiatry, Geisel School of Medicine, Dartmouth College, Lebanon, NH 03766 USA

**Keywords:** Psychology, Biomarkers, Risk factors

## Abstract

Generalized anxiety disorder (GAD) and major depressive disorder (MDD) are highly prevalent and impairing problems, but frequently go undetected, leading to substantial treatment delays. Electronic health records (EHRs) collect a great deal of biometric markers and patient characteristics that could foster the detection of GAD and MDD in primary care settings. We approached the problem of predicting MDD and GAD using a novel machine learning pipeline to re-analyze data from an observational study. The pipeline constitutes an ensemble of algorithmically distinct machine learning methods, including deep learning. A sample of 4,184 undergraduate students completed the study, undergoing a general health screening and completing a psychiatric assessment for MDD and GAD. After explicitly excluding all psychiatric information, 59 biomedical and demographic features from the general health survey in addition to a set of engineered features were used for model training. We assessed the model's performance on a held-out test set and found an AUC of 0.73 (sensitivity: 0.66, specificity: 0.7) and 0.67 (sensitivity: 0.55, specificity: 0.7) for GAD, and MDD, respectively. Additionally, we used advanced techniques (SHAP values) to illuminate which features had the greatest impact on prediction for each disease. The top predictive features for MDD were being satisfied with living conditions and having public health insurance. The top predictive features for GAD were vaccinations being up to date and marijuana use. Our results indicate moderate predictive performance for the application of machine learning methods in detection of GAD and MDD based on EHR data. By identifying important predictors of GAD and MDD, these results may be used in future research to aid in the early detection of MDD and GAD.

## Introduction

Major depressive disorder (MDD) and generalized anxiety disorder (GAD) are prevalent psychiatric disorders that affect 16.2% and 13.3% of U.S. individuals, respectively, over their lifetimes^1,2^. MDD is the leading cause of disability worldwide^3,4^, and anxiety disorders are the sixth leading cause of disability^5^. MDD is characterized by persistent low mood, associated with disturbances with sleep, motivation, energy, appetite, and suicidal thoughts^6^. GAD represents a persistent, uncontrollable pattern of worry occurring in multiple domains of an individual’s life^7^. Left untreated, these syndromes often have devastating consequences for affected individuals, their families, and communities^8,9^.

Both MDD and GAD are prevalent in the college population. In a 2015 study, 23% of surveyed college students reported moderate to severe depressive symptoms^10^. Similarly, a 2019 study showed a 20% prevalence of GAD among college students in 2016, representing a 100% increase since 2008^11^. These syndromes negatively impact multiple domains of an individual’s functioning, and for college students, this may include interference with class attendance and learning retention^12^. Research among college students found that students with depression are more likely to report drinking-related harms and alcohol abuse^13^.

Two major challenges in adequately addressing MDD and GAD are identifying affected individuals and ensuring appropriate and timely treatment. Because MDD and GAD symptoms are internally experienced, MDD and GAD often go undetected^14–16^. There is an estimated 6 year and 14 year delay between disease onset and intervention for MDD and GAD, respectively, during which time the disease may increase in severity, lowering student quality of life^17,18^.

Early detection and diagnosis is paramount to understanding and addressing mental illness on a populational level. With the rise in electronic health records (EHRs), spurred by initiatives like the Health Information Technology Act (Rights (OCR), 2009), there is increasing potential for addressing previously intractable clinical questions using computational analysis of large data sets. Multiple studies show promise in this area^19–23^.

A 2011 study by Trinh et al.^19^ found that an EHR billing diagnosis of “depression” can serve as an effective proxy for identifying clinical depression. Although this study did not exploit advanced statistical models, it demonstrated prediction of psychiatric pathology using structured EHR data, albeit the clinical utility of these predictive models is questionable given that the predictors used were closely related to outcome. Perlis et al.^20^ found improvements in prediction of MDD using unstructured clinical narrative features (extracted with NLP) and billing code data, compared with using billing code data alone. A more recent 2019 study by Wang et al.^21^ utilized machine learning techniques for prediction of postpartum depression (PPD). The predictors were extracted from the EHR and the model ended up with a good predictive accuracy. Features found to be significant included *depression*, *anxiety*, *use of antidepressant drugs*, and *pain diagnoses*. Geraci et al.^22^ used data extracted from psychiatric clinical texts to predict a diagnosis of depression, including both structured or unstructured psychiatric diagnoses. Huang et al.^23^ exploit multiple structured features to predict depression, including diagnostic codes and patient prescriptions, which could include psychiatric medications.

Although promising early directions, a common limitation in these studies^19–23^ is the use of features highly interdependent with MDD, including psychiatric billing codes or unstructured notes, likely containing explicit diagnostic information. This presents as a major limitation to the potential utility of using these prior studies to close the onset to treatment gap among those with MDD and GAD. In particular, diagnostic codes could only be obtained from those whose MDD and GAD would have already been detected.

Based on the limitations of prior studies that utilized psychiatric features to predict GAD and MDD, our study utilized an EHR dataset containing biometric and demographic data from 4184 undergraduate students. Excluding all psychiatric features, we approach the problem of identification and diagnosis using a novel machine learning pipeline developed for the purpose of this study. The pipeline constitutes an ensemble of multiple algorithmically distinct machine learning methods, including deep learning methods. We trained the model to predict psychiatric illness using varied non-psychiatric input features such as blood pressure, heart rate, housing status, and public insurance. This is to say, unlike all prior studies, we did not use any psychiatric information in predicting diagnosis of GAD or MDD. We hypothesized that using such biomedical data, we could predict MDD and GAD with a level of certainty above chance. Our primary aim was to identify important predictors for GAD and MDD risk.

## Methods

### Participants

Four thousand one hundred and eighty four undergraduate students from the University of Nice Sophia-Antipolis underwent a basic medical examination and participated in the current study. All data was publicly available on Dryad and completely de-identified and therefore this research does not meet the federal definition for human subjects research. Additionally, according to the original study, the National Data Protection Authority (NCIL) approved the study^24^. The methods of the study carried out in France were in accordance with the laws of non-interventional clinical research^24^. Due to this being an observational study in compliance with laws that regulate non-interventional clinical research in France (articles L.1121-1 and R.1121-2 of the Public Health Code), informed consent was not required^24^. Additionally, this study received institutional exemption from the Committee for the Protection of Human Subjects at Dartmouth College. These students were 57.4% female and 42.6% male and their ages were split into four categories: less than 18, 18, 19 and 20 or older. The distribution among these categories was as follows: 5%, 36%, 28% and 31%. The outcomes of interest, MDD and GAD, had base rates of 12% and 8% respectively^24^.

### Features

A total of 59 features were used including binary, ordinal and continuous variables. Specifically, features included age (4 levels: under 18, 18, 19, over 20), gender, French nationality, field of study, year of university, learning disabilities, difficulty memorizing lessons, professional objective (whether the student indicated an objective), informed about opportunities (whether the student indicated that they felt informed about opportunities at the university), satisfied with living conditions, living with a partner/child, parental home, having only one parent, at least one parent unemployed, siblings (yes/no), long commute, mode of transportation, financial difficulties, grant (yes/no), additional income (yes/no), public health insurance, private health insurance, universal health coverage, irregular rhythm of meals, unbalanced meals, eating junk food, on a diet, irregular rhythm or unbalanced meals, physical activity (3 levels: none, occasional, regular) , physical activity (2 levels: none or occasional, regular), weight (kg), height (cm), overweight and obesity, systolic blood pressure (mmHg), diastolic blood pressure (mmHg), prehypertension or hypertension, heart rate (bpm), abnormal heart rate, distant visual acuity of right eye (score/10), distant visual acuity of left eye (score/10), close visual acuity of right eye (score/10), close visual acuity of left eye (score/10), decreased in distant visual acuity, decreased in close visual acuity, urinalysis (glycosuria), urinalysis (proteinuria), urinalysis (hematuria), urinalysis (leukocyturia), urinalysis (positive nitrite test), abnormal urinalysis, vaccination up to date, control examination needed (whether the student needed a follow-up for any reason), cigarette smoker (5 levels: none, occasional, regular, frequent, heavy), cigarette smoker (3 levels: no, frequent, occasional), drinker (3 levels: no, occasional, regular), drinker (2 levels: no or occasional, regular or heavy), binge drinking, marijuana use, other recreational drugs.

### Psychiatric diagnoses

The outcomes of interest were MDD and GAD. MDD and GAD were each assessed in a multi-stage process. The first stage included a screening questionnaire that assessed four hallmark symptoms of MDD (anhedonia, loss of energy/fatigue, changes in activity and depressed mood) and four hallmark symptoms of GAD (excessive worry, restlessness, fatigue, and irritability). If the assessment indicated possible presence of either disorder (positive answer to two of the four categories), the participants were assessed for full Diagnostic and Statistical Manual of Mental Disorders Fourth Edition (DSM IV) criteria by a medical provider^24^.

### Data preprocessing

The preprocessing pipeline included creating dummy variables for ordinal outcomes, normalizing continuous variables, and single imputation for missing values using a Bayesian Ridge approach across features. A total of 20 of the 59 variables included NA values and the percentage missing ranged from < 1% to 36%. Total missingness was 5% and median missingness across all variables was 0%.

To enhance our model, we used feature engineering, informed by domain specific biomedical knowledge. Feature engineering as used in our study refers to the combination of distinct features into new “engineered” features, which have domain specific meaning and utility. Previous research has shown feature engineering to improve machine learning model performance^25,26^. By combining existing features, we created and used (1) Body Mass Index (BMI)^27^, (2) Mean Arterial Pressure (MAP)^28^, and (3) Pulse Pressure^29^. BMI is a function of an individual's height and weight. MAP and pulse pressure are clinically meaningful combinations of diastolic and systolic blood pressure.

### Data analysis

**Figure 1:**
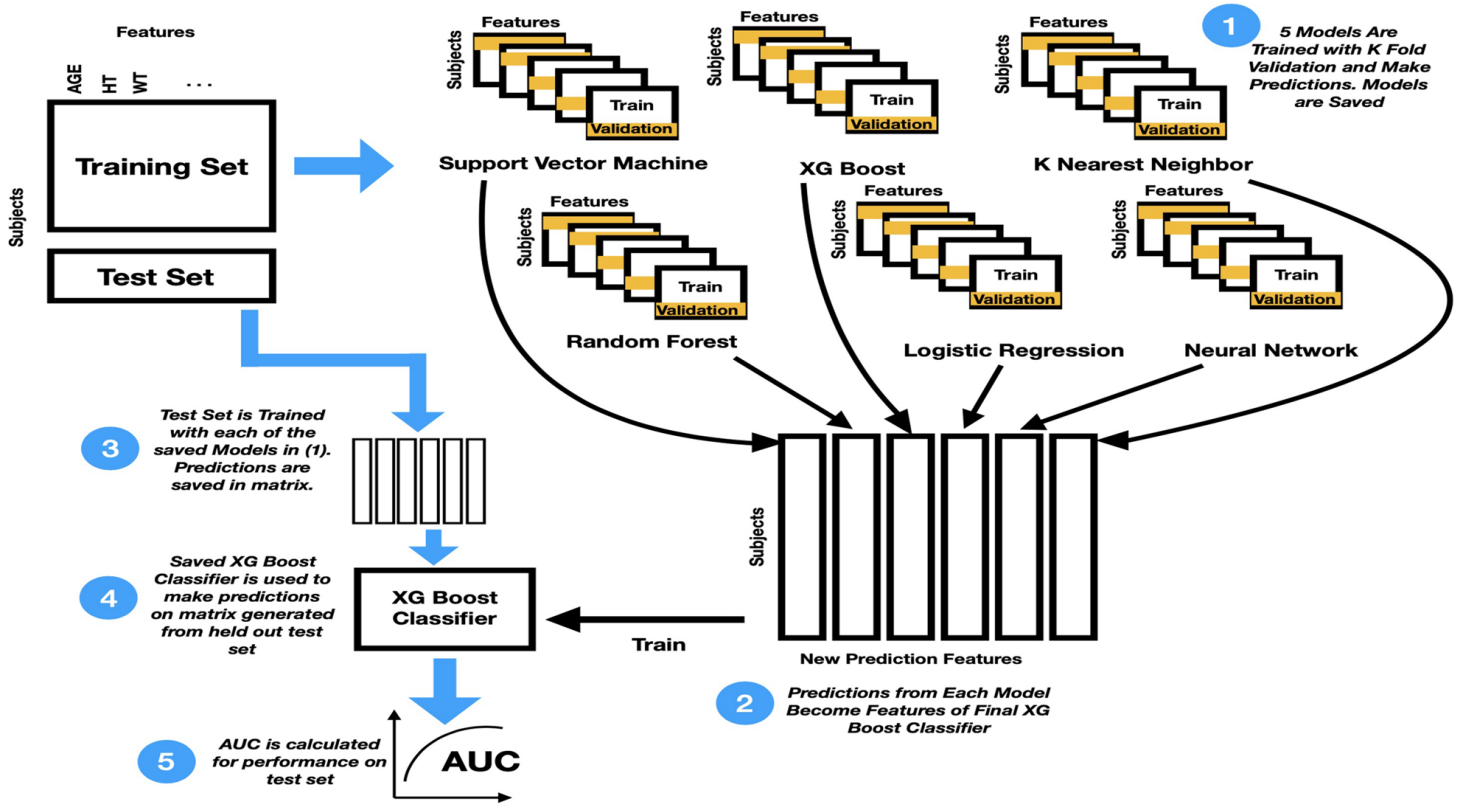
This is the pipeline used to train the machine learning models and generate predictions. The training set is sent through fivefold training for each model type to generate a prediction for each training sample. These predictions are then used to train a higher level model to predict a final outcome given the predictions from the fivefold training. Each of the 6 models from each fold then predicts on the held out test set and the average prediction for the probability of depression is stored. The higher level model then makes final predictions on the held out test set.

The first step of analysis was dividing the data into 70% training (*N* = 2929) and 30% (*N* = 1255) held out testing (see Fig. 1). The held out test set remained unseen throughout model training and was never used for hyperparameter tuning. The machine learning pipeline included six algorithmically unique machine learning classifiers to inform final predictions. These classifiers were XGBoost, Random Forest, Support Vector Machine, K-nearest-neighbors and a neural network tuned using Bayesian hyperparameter optimization. A fivefold validation technique was used to train each model. This allowed for each model type (e.g., logistic regression) to make one prediction for each subject in the training set. These predictions were saved to be used as inputs to a “higher level” model that would eventually make final predictions.

The aforementioned “higher level” model was an XGBoost classifier which was trained, again, using fivefold validation, on the predictions of the original 6 models. Essentially, each “lower level” model made a prediction (i.e. probability of MDD) for each subject and the higher level model decided which model’s predictions were most informative based on the true outcome. Using this information, the higher-level model made a final estimation for the probability of the outcome of interest.

These models were then used to make predictions on the held out test set to ensure there was no overfitting and that the results were meaningful and generalizable. To create the prediction matrix on the held out test set, all 5 saved models for each machine learning method made predictions on each subject. The predictions for each model type were then averaged and filled into the prediction matrix. The high level XGboost model then made final predictions. The area under the receiver operating characteristic curve (AUC) is a measure of how well the model can effectively distinguish between psychiatric diagnosis, reflecting the model performance in optimizing across both sensitivity and specificity. To guide interpretation of the results, please note that an AUC = 0.58 represents a small effect size, AUC = 0.69 represents a medium effect size, and AUC = 0.79 represents a large effect size, based on conversions to Cohen’s d values of 0.2, 0.5, and 0.8 respectively^30^. This pipeline was used twice, once with the outcome being GAD and once with the outcome being MDD.

### Model explainability

SHAP (Shapley Additive Explanations) scores were utilized calculate and visualize feature importance this complex model^31^. The SHAP kernel explainer allows for a user to input data and a prediction function and it will return the relative importance for each feature for each subject. The prediction function, in this case, simply took the input data and utilized the trained models from the pipeline to make predictions. These predictions were then averaged across the lower level models and fed into the upper level model. The upper level model returned the final prediction for each subject. With this setup, the kernel explainer would return the SHAP values for each of the features from the original input data based on how it informed the entire pipeline’s prediction.

## Results

### Predictive performance

**Figure 2:**
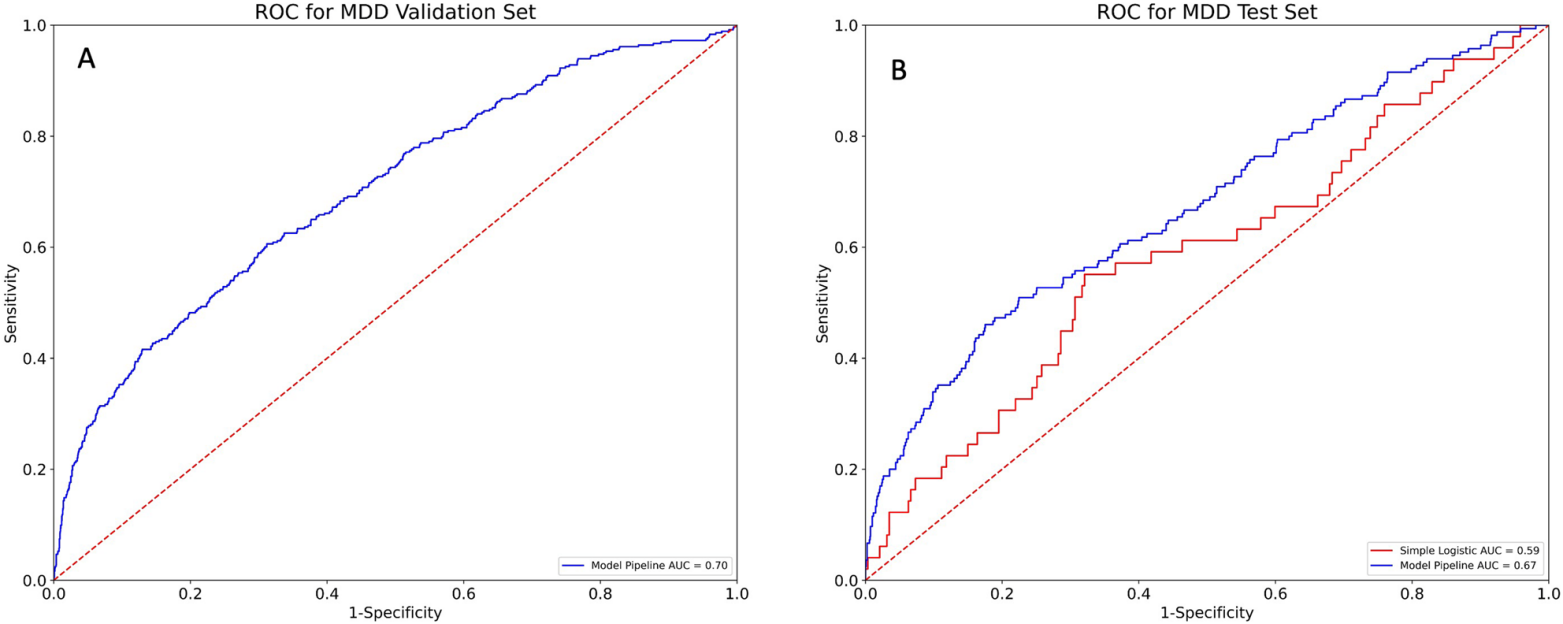
(**A**) AUC for prediction of MDD in the training set. (**B**) AUC for the prediction of depression in the held-out test set using both a simple logistic regression and our novel pipeline. These curves show the sensitivity and specificity at different thresholds for prediction.

**Figure 3:**
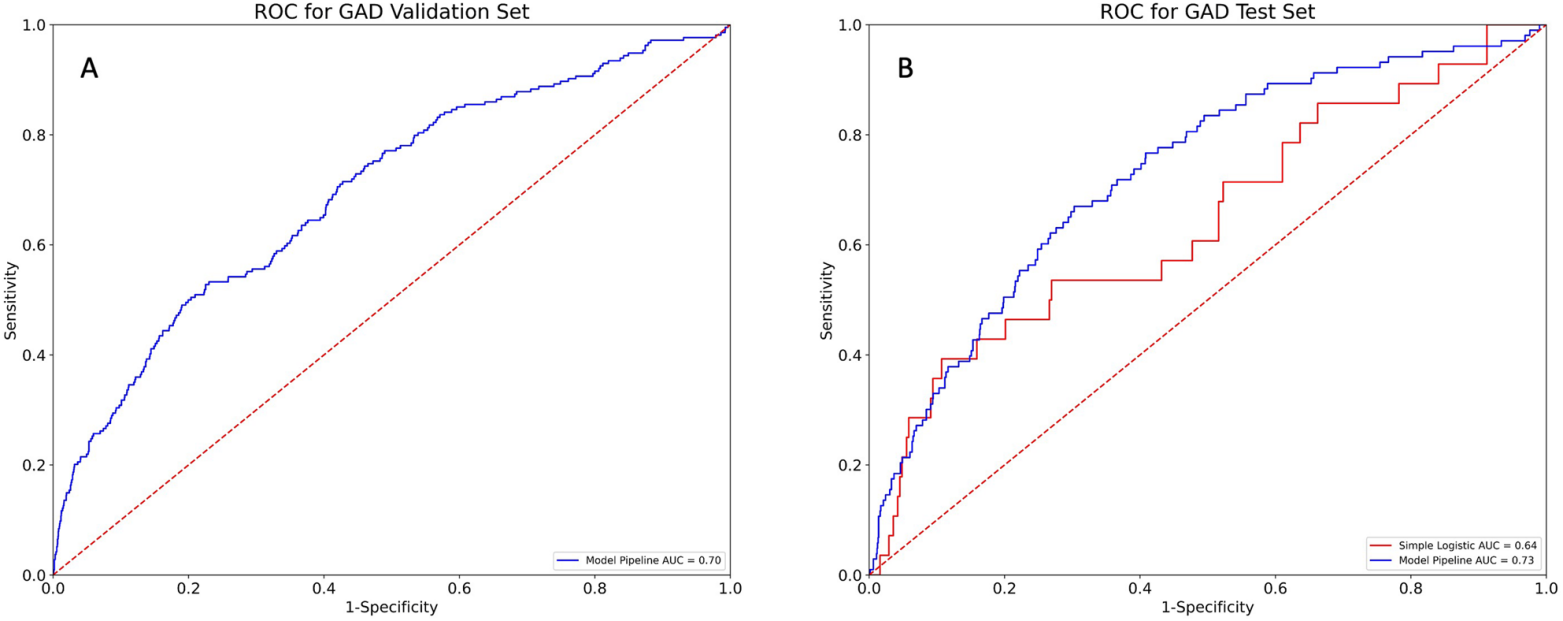
(**A**) AUC for prediction of GAD in the training set. (**B**) AUC for the prediction of anxiety in the held-out test set using both a simple logistic regression and our novel pipeline for prediction. These curves show the sensitivity and specificity at different thresholds for prediction.

The main results of this study are two-fold, the first is the prediction accuracy of the stacked machine learning models and the second is the important features driving those predictions. The validation and test-set AUC for MDD (see Fig. 2) and GAD (see Fig. 3) were (0.70, 0.67) and (0.70, 0.73) respectively. Thus, the ensemble model could predict diagnosis of MDD and GAD well above chance and with a medium effect size. Additionally, when compared to a simple standard logistic regression as run in the original study, the AUCs of the complex machine learning models were increased, on average, by 0.08 (Figs. 2B, 3B). Given the AUC curve of the model, we can choose thresholds with higher sensitivity at the detriment of specificity. Given the non-invasive nature of secondary screening for each of these illnesses, it seems reasonable to allow a soft threshold for further diagnosis. Specifically, for MDD, the sensitivity and specificity were 55% and 70% respectively. Additionally, the positive predictive value was 20% and the negative predictive value was 92%. For GAD, the sensitivity and specificity were 70% and 66% respectively. The positive predictive value was 16% and the negative predictive value was 96%.

### Model explainability

**Figure 4:**
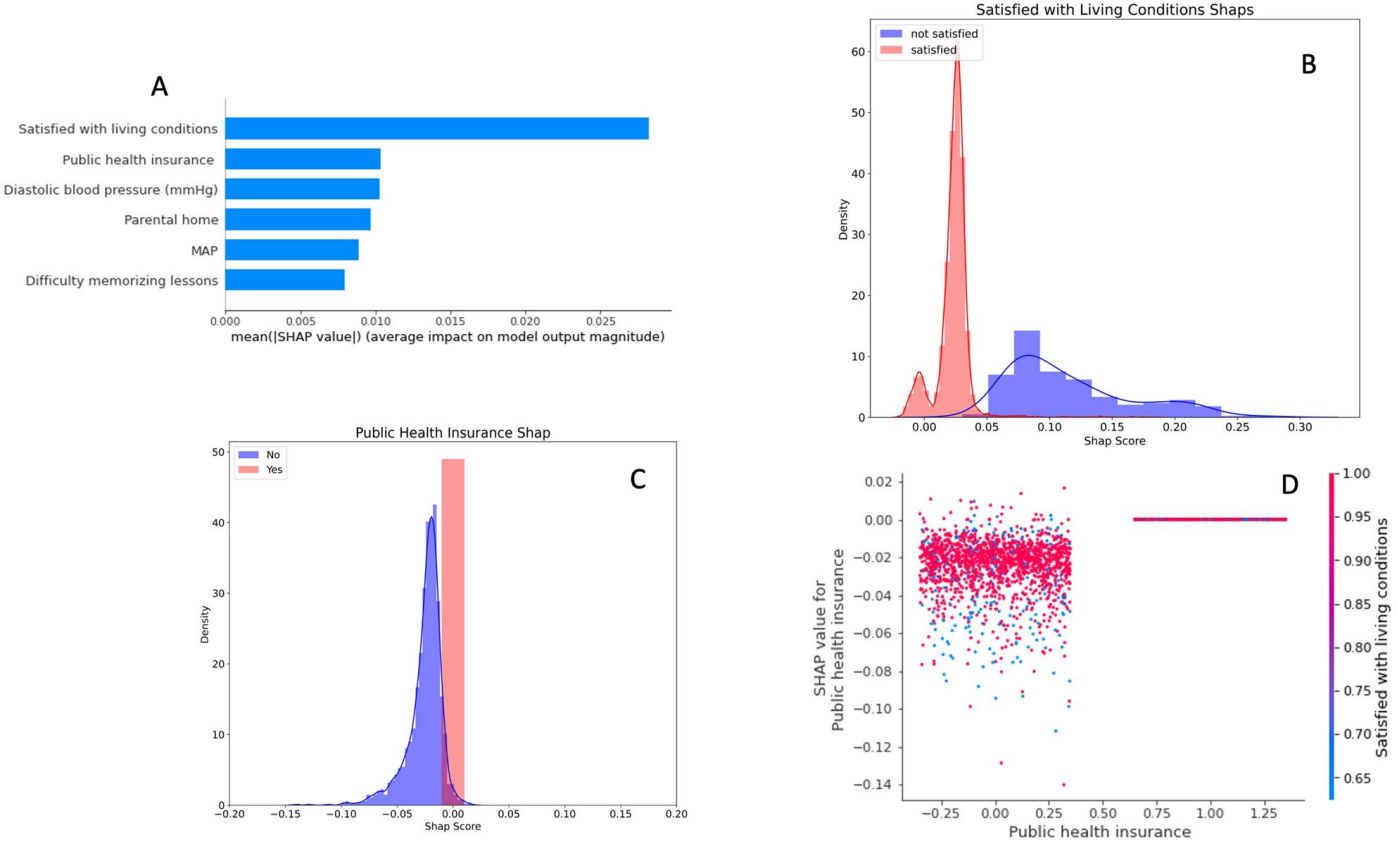
(**A**) This plot shows the top six most important features for predicting MDD. This is displayed as the mean of the absolute value of SHAP scores across all subjects for that given feature. A higher SHAP value indicates that the feature was important in informing the models prediction. (**B**) This plot displays the density distribution of SHAP values for the top performing feature in predicting depression. (**C**) This plot also displays the density distribution of SHAP values for the second most important feature in predicting MDD. Positive SHAP score indicates that the feature was indicative of the subject having MDD. (**D**) This is an interaction plot showing the effect of two features working together to inform the model. Here it is apparent that when a student does not have public health insurance, living conditions can partially inform prediction.

**Figure 5:**
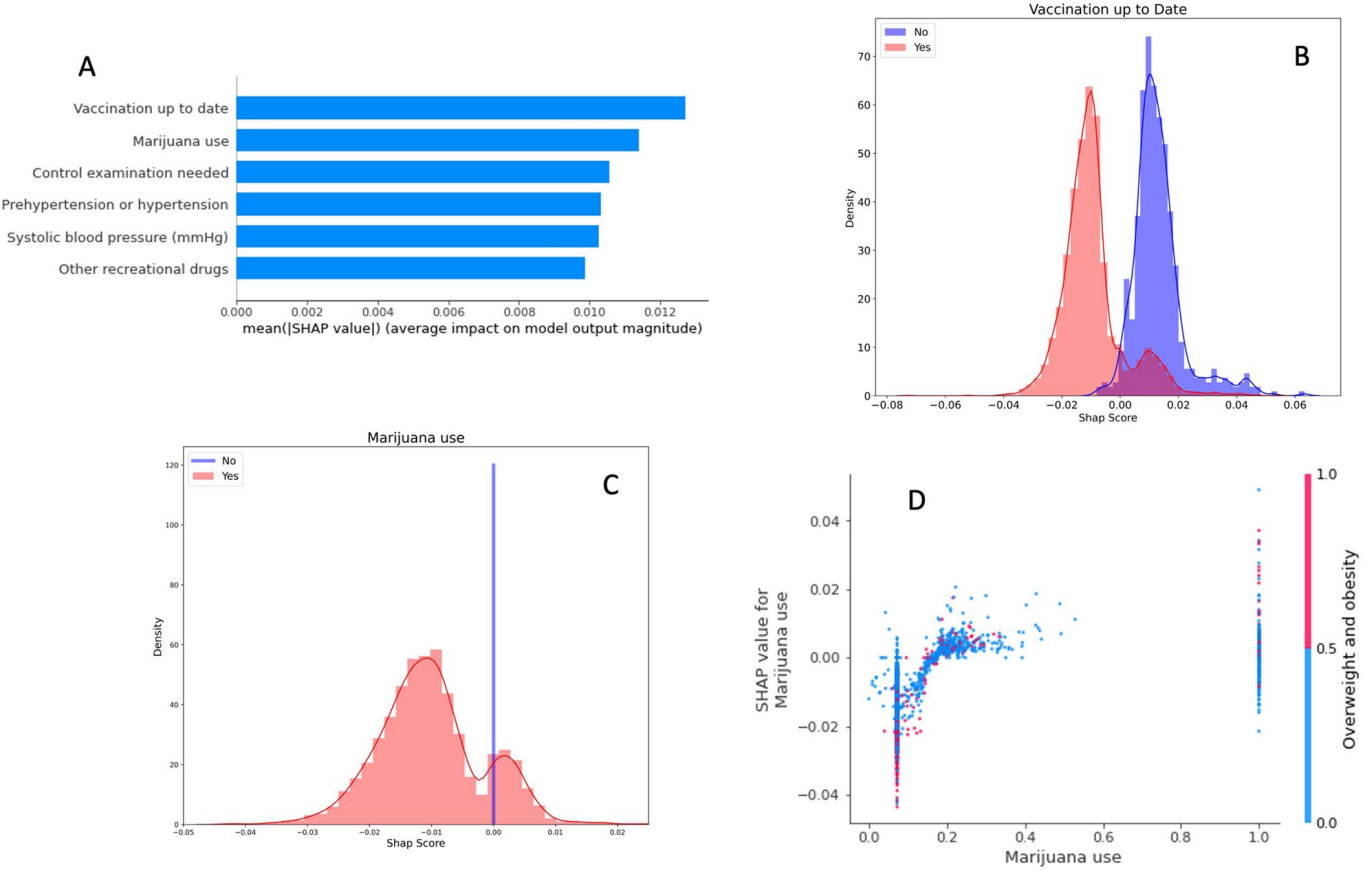
(**A**) This plot shows the top six most important features for predicting GAD. This is displayed as the mean of the absolute value of SHAP scores across all subjects for that given feature. A higher SHAP value indicates that the feature was important in informing the models prediction. (**B**) This plot displays the density distribution of SHAP values for the top performing feature in predicting GAD. (**C**) This plot also displays the density distribution of SHAP values for the second most important feature in predicting GAD. Positive SHAP score indicates that the feature was indicative of the subject having GAD. (**D**) This is an interaction plot showing the effect of two features working together to inform the model. Here it is apparent that marijuana use is more predictive of GAD in overweight students.

The second and arguably more important set of results are the important features and how they inform predictions (Figs. 4, 5). The top features (Figs. 4A, 5A) are the most informative to the model but it is important to note that the impact of features on the outcome was distributed across a large number of features (i.e. the SHAP values for top features were small). This is likely indicative of the complex and heterogenous nature of the disease. To ascertain either MDD or GAD status, it requires a not just a singular important predictor but rather a combination of features and feature interactions to accurately assess the disease state. This exemplifies the necessity for complex models to disentangle the relationships between variables and characterize and assess the disease in any given person.

*MDD (See Fig. *4*)* The most important feature driving the prediction of MDD was whether the student was satisfied with their living conditions (4b). High diastolic blood pressure was also indicative of MDD and having public health insurance indicated, for the most part, non-MDD status (4c). In order, living in a parental home, mean arterial pressure and difficulty memorizing lessons made up the remaining important predictors from the top six. Additionally, after further assessing these top features, it was noted that many of them were predictive as part of two-way interactions, such that the relationship between a predictor and an outcome is conditional on another predictor. As seen in Fig. 4D, typically individuals without public health insurance had lower predictions of MDD, but the extent was conditional on whether they were satisfied with their living conditions. Those who were satisfied with their living conditions seemed to be slightly more informative in telling the model that MDD was not apparent.

*GAD (See Fig. *5*)* The most important predictor for GAD was having up to date vaccinations (4b). Another similar and important variable for prediction was the necessity for a control examination. This was essentially a binary indicator for whether or not the student needed to return to the doctor for something unrelated to the psychiatric outcome. The second most important predictor was marijuana use although the effect of this variable on model prediction was clearly impacted by interactions with other subject characteristics (4c). The remaining top six most important predictors were, in order, hypertension or prehypertension, systolic blood pressure and the use of other recreational drugs. These features, overall, were all much closer in importance than in MDD. This further indicated the model’s reliance on all features, not just one important predictor. Again, there were very clear two-way interactions between variables when the model was making predictions. Smoking marijuana was clearly more indicative of predicted GAD if the individual was overweight or obese (4d). Other interactions included systolic blood pressure with prehypertension and hypertension and the necessity of a control examination with gender.

## Discussion

Our objective was to evaluate the importance and effectiveness of standard clinical data on the prediction of MDD or GAD. We used state-of-the-art novel machine learning methodologies to make predictions. Additionally, SHAP values were generated to explain and clinically validate our findings. We trained our model with > 2500 participants and assessed the model's performance on a held-out test set. Although our accuracy metrics are comparable to previous studies predicting psychiatric outcomes, ours is unique in its primary reliance on routine biomedical and demographic features, rather than features with a known correlation to psychiatric outcomes. Previous studies that have looked at EHR to detect MDD have had the significant limitation of including predictive variables that would nullify the clinical utility of the model by relying on features that are directly indicative of known psychiatric illness (e.g. including psychiatric billing codes, which are based upon clinician diagnosis). Thus, this study is the first known study to predict MDD and GAD using EHR data with potential for predictive validity in detecting unknown psychiatric diagnoses.

Studies using magnetic resonance imaging (MRI) have been able to achieve slightly higher predictive performances ranging from 67 to 94%^32^. Nevertheless, perhaps due to the considerable expense of collecting MRI data, a common limitation of these was their small sample sizes. These studies also had considerable range in performance, and the due to their small sample sizes the results are highly inconsistent^33^. Moreover, using MRI to predict MDD is unrealistic when there is no other reason to justify an MRI, especially in an otherwise physically healthy college-age patient.

In addition to the complex machine learning approach and our carefully curated feature set, we are providing insights to the complex clinical appearance of MDD. Our pipeline, using SHAP values to visualize feature importance, provides not only the outcome prediction but the possible characteristics that a physician can identify when making a decision. These characteristics including mean arterial pressure, blood pressure, markers for low SES and general health markers have been shown to be previously associated with depression and anxiety^34,35^. Of note, despite the potential for these relationships between indicators of low SES and MDD and GAD, this study cannot rule out that this was based on biased physician diagnoses.

In further investigation of the predictors for generalized anxiety disorder, vaccination status may be reflective of overall poorer health outcomes in individuals with GAD^36^. Regarding the “marijuana use”, prior research demonstrates high comorbidity between anxiety disorders and substance use disorders^37^. With regard to the most important features driving major depressive disorder, there is research supporting overall poorer life satisfaction in individuals with MDD^38^, which may certainly include dissatisfaction with living conditions. Low interest and energy, DSM criteria for MDD, may contribute to difficulties maintaining satisfactory living conditions. Robust research to date indicates that individuals of lower socioeconomic status are more likely to have MDD^39^. “Difficulty memorizing lessons” may be related to concentration difficulties, also identified by the DSM as a clinical feature of MDD. An additional top predictive feature for both MDD and GAD is hypertension. Research to date corroborates this finding by demonstrating that individuals with either MDD or GAD are more likely to have hypertension^40,41^.

This information has the potential to allow health care providers to make informed recommendations for further screening regardless of whether the patient discusses or even recognizes his or her symptoms. This is important because as previously mentioned, it can take on average 6 or 14 years from onset of illness until diagnosis for MDD and GAD respectively^17^. Our study is one of the first of its kind to tackle this issue by not relying on previous psychiatric diagnoses or expensive imagine techniques to capture the disease in an early stage.

This study has several important limitations which deserve mention. One is that the original screening for the outcomes of MDD and GAD may not have captured all cases within the population. This, in addition to the study population, limits the generalizability of the results. Our dataset comes from French college aged students, who likely have baseline differences from other populations with psychiatric illness. Despite this limitation, our study still serves to show the predictive ability of mainly non-psychiatric variables for psychiatric illness. Such variables, further analyzed individually for their connection to psychiatric pathology, may prove the basis of further research. Another limitation of our study, which is fairly ubiquitous in mental health research is the low prevalence of anxiety and depression in our study population, as well as our sample size. Although this is a limitation in many studies of psychiatric nature, we were able to enhance our predictive power using a stacked ensemble model pipeline. Additionally, the lack of qualitative information (i.e., severity, subtype, etc.) regarding mental health diagnoses was not available to allow for a severity prediction analysis. Thus, future research should examine the potential for these important predictor to predict severity and subtype of MDD and GAD.

This research is an important step in the direction towards identifying potentially difficult to diagnose illnesses with readily available and easy to obtain information. Our tool, using an optimal sensitivity/specificity split would be able to capture two out of every three subjects with GAD and one out of two MDD cases while only incurring a 30% false positive rate. Because there are detrimental outcomes to both the patient and provider in a false positive, looking at the efficacy of case identification while requiring 70% specificity gives a reasonable idea of how many cases would be captured if this model were to be deployed in a clinical setting. These findings have shown promise on multiple fronts: Ability to use easy to obtain information to inform possible detection of MDD and GAD, further understanding of the demographic and biological characteristics associated with illness, and both the success and necessity for computational tools to inform psychological medicine. We believe, given a larger and more heterogeneous sample, this modeling technique could be used to elucidate the drivers of psychological illness and provide a tool that indicates the necessity of treatment with high precision and accuracy.

## Supplementary Information

Supplementary Information.
