# Supplementary material for: Predictive modeling of depression and anxiety using electronic health records and a novel machine learning approach with artificial intelligence
Source: Sci Rep. 2021 Jan 21;11:1980. doi: 10.1038/s41598-021-81368-4 (PMC7820000; doi:10.1038/s41598-021-81368-4)
Supplement: Supplementary file 1 — Supplementary Information. [file 41598_2021_81368_MOESM1_ESM.docx]

Predictive Modeling of Depression and Anxiety using Electronic Health Records and a Novel Machine Learning Approach with Artificial Intelligence

Matthew D. Nemesure, BS*^1,2^; Michael V. Heinz, MD ^1,3^; Raphael Huang^1^, and Nicholas C. Jacobson; PhD^1,2,4,5^


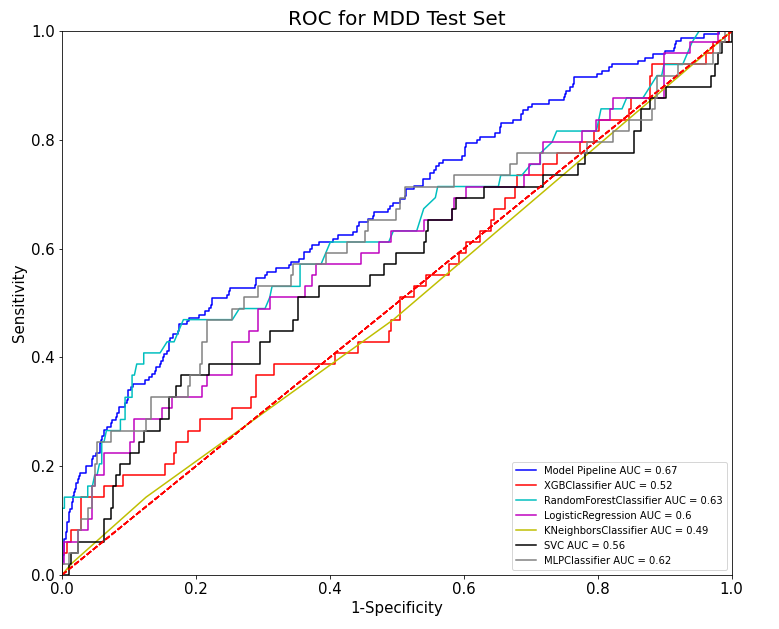


Figure 1

Figure 1: Final AUC for predicting MDD in the held out test set for each individual model and the ensemble model.


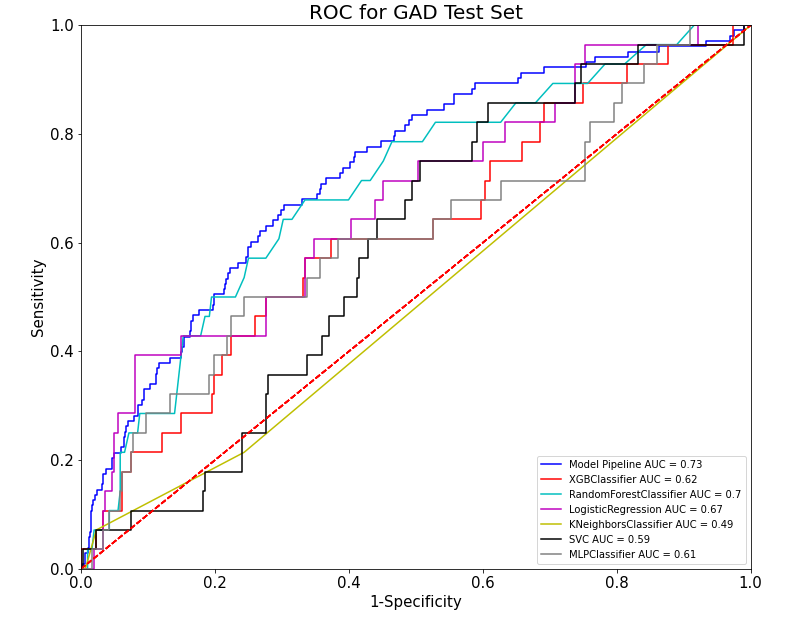


Figure 2

Figure 2: Final AUC for predicting GAD in the held out test set for each individual model and the ensemble model.

Github link: <https://github.com/mnemesure/MDD_GAD_EHR>

Matplotlib:

Matplotlib: Thomas A Caswell, Michael Droettboom, Antony Lee, John Hunter, Elliott Sales de Andrade, Eric Firing, … Paul Ivanov. (2020, November 12). matplotlib/matplotlib: REL: v3.3.3 (Version v3.3.3). Zenodo. http://doi.org/10.5281/zenodo.4268928

Seaborn: Michael Waskom, Olga Botvinnik, Maoz Gelbart, Joel Ostblom, Paul Hobson, Saulius Lukauskas, … Thomas Brunner. (2020, September 8). mwaskom/seaborn: v0.11.0 (Sepetmber 2020) (Version v0.11.0). Zenodo. http://doi.org/10.5281/zenodo.4019146
